# Development of Renal Failure without Proteinuria in a Patient with Monoclonal Gammopathy of Undetermined Significance: An Unusual Presentation of AL Kappa Amyloidosis

**DOI:** 10.1155/2012/573650

**Published:** 2012-10-02

**Authors:** Yijuan Sun, Amarpreet Sandhu, Darlene Gabaldon, Jonathan Danaraj, Karen S. Servilla, Antonios H. Tzamaloukas

**Affiliations:** Division of Nephrology, Raymond G. Murphy Veterans Affairs Medical Center, The University of New Mexico School of Medicine and VA Medical Center (111C), 1501 San Pedro SE, Albuquerque, NM 87108, USA

## Abstract

AL amyloidosis complicating monoclonal gammopathy of undetermined significance (MGUS) has usually a predominant glomerular deposition of lambda light chain. Heavy proteinuria is one of its cardinal manifestations. A 78-year-old man with a 9-year history of IgG kappa light-chain-MGUS and normal urine protein excretion developed severe renal failure. Serum levels of kappa light chain and serum IgG had been stable while proteinuria was absent throughout the nine-year period. For the first eight years, he had stable stage III chronic kidney disease attributed to bladder outlet obstruction secondary to prostatic malignancy. In the last year, he developed progressive serum creatinine elevation, without any increase in the serum or urine levels of paraproteins or any sign of malignancy. Renal ultrasound and furosemide renogram showed no evidence of urinary obstruction. Renal biopsy revealed AL amyloidosis, with reactivity exclusive for kappa light chains, affecting predominantly the vessels and the interstitium. Glomerular involvement was minimal. Melphalan and prednisone were initiated. However, renal function continues deteriorating. Deposition of AL kappa amyloidosis developing during the course of MGUS predominantly in the wall of the renal vessels and the renal interstitium, while the involvement of the glomeruli is minimal, leads to progressive renal failure and absence of proteinuria. Renal biopsy is required to detect both the presence and the sites of deposition of renal AL kappa light chain amyloidosis.

## 1. Introduction

The term monoclonal gammopathy of undetermined significance (MGUS) covers a spectrum of conditions characterized by production of a monoclonal immunoglobulin. The characteristic features of MGUS are a plasma concentration of the monoclonal protein less than 3 gm/dL, plasma cells representing less than 10% of all bone marrow cells, and absence of hypercalcemia, elevated serum creatinine, anemia (above 1.5 mg/dL), lytic bone lesions or severe symptomatic osteoporosis, and B-cell lymphoproliferative malignancy [1]. 

The prevalence of MGUS increases with age. In Olmsted County, MN, USA, 3.2% of the subjects 50 years old or older and 5.3% of the subjects 70 years old or older had MGUS [2]. A variety of life-threatening conditions may complicate MGUS. The risk of development of one of these conditions is approximately 1% per year of followup [3]. The list of conditions complicating MGUS includes multiple myeloma, Waldenstrom's macroglobulinemia, other lymphoproliferative malignancies such as chronic lymphocytic leukemia and IgM lymphoma, other hematologic malignancies, AL amyloidosis, chronic inflammatory demyelinating polyradiculopathy, autonomic neuropathy, osteoporosis, fractures of vertebrae and hips, liver and kidney transplants, hypercalcemia, and urticaria [3–5]. 

Renal disease, with a variety of histological pictures, complicates frequently the course of MGUS [6]. A distinct form of MGUS, light-chain-monoclonal gammopathy of undetermined significance (LC-MGUS), with a similar frequency of renal disease as the classical MGUS entity, has also been recognized [7]. AL amyloidosis with renal deposits of light chains is one of the conditions which may complicate MGUS [6]. Nephrotic syndrome is the most frequent clinical presentation of this type of amyloidosis [6]. We present a patient who developed renal AL kappa amyloidosis manifested by progressive renal failure without proteinuria after several years of MGUS with stable paraprotein levels and renal function. Absence of extensive amyloid deposits in the glomeruli was the apparent reason for the absence of proteinuria in this patient. 

## 2. Case Report

A 78-year-old Caucasian man presented with stage V chronic kidney disease (CKD) in June 2012. MGUS had been diagnosed in early 2003. At that time, serum protein immunoelectrophoresis revealed a paraprotein with a concentration of 1.4 g/dL and characterized as IgG kappa by immunofixation; serum IgG was 2,263 mg/dL (normal range 694–1618 mg/dL); hemogram and serum IgA, IgM, albumin, calcium, and uric acid levels were normal; serum creatinine was 1.2 mg/dL; and urinalysis had no proteinuria. Bone marrow examination was not performed. In August 2003, he had a radical suprapubic prostatectomy for prostatic adenocarcinoma. He subsequently developed urinary incontinence with several bouts of urinary tract infection and stage III CKD with serum creatinine levels between 1.2 and 1.5 mg/dL. In November 2004, he had surgery for creation of artificial urinary sphincter. 

Between 2003 and 2012, his renal function remained stable. Serum levels of the paraprotein fluctuated between 1.4 and 2.3 g/dL, while serum IgG levels fluctuated between 1.4 and 2.4 g/dL. Serum-free kappa chain fluctuated between 38.3 and 258.7 mg/dL (normal range 3.3–19.4 mg/dL), and kappa/lambda ratio fluctuated between 4.20 and 8.36 (normal range 0.26–1.65). Blood hemoglobin, white cell and platelet counts, and serum calcium and albumin remained within normal limits. Skeletal surveys showed no lytic bone lesions, while dual-energy X-ray absorptiometry (DEXA) studies showed osteopenia, but no osteoporosis.

Serum creatinine was 2.28 mg/dL in January 2012 and increased progressively afterwards. He had not been on antihypertensive medications in the past and had no elevations of his blood pressure after the start of the rise in serum creatinine. Small amounts of IgG-kappa paraprotein (0.3–10.3 mg/dL) were repeatedly detected in the urine. However, proteinuria, evaluated numerous times by urinalysis, spot urine protein to creatinine ratios and urine protein electrophoresis, had consistently remained in the normal range, even during episodes of urinary tract infection when pyuria and hematuria were present. Only one spot sample done while serum creatinine was 5.44 mg/dL in July 2012 had a small elevation of the urine protein/creatinine ratio to 0.3 mg/mg (normal values ≤0.2 mg/mg). Subsequent determinations of urine protein/creatinine ratio were all <0.2 mg/mg. Ultrasonography of kidneys, ureters and urinary bladder, and furosemide renogram did not disclose a urinary tract obstruction. A percutaneous kidney biopsy was performed in June 2012. At that time, serum creatinine was 3.86 mg/dL.

**Figure 1:**
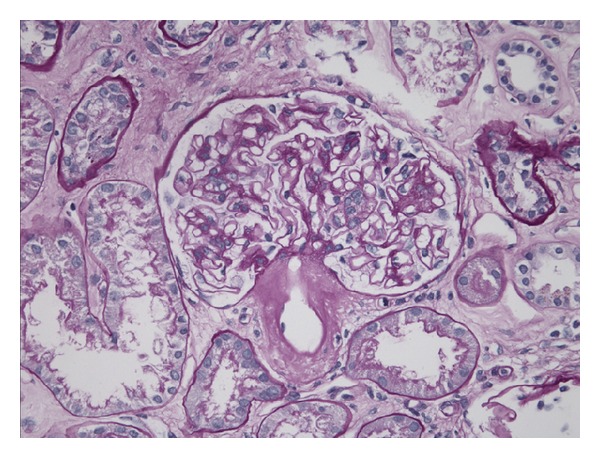
Light microscopy. Adjacent efferent arteriole with significant deposits of amorphous eosinophilic material; glomerulus with same limited and segmental deposits in its wall; interstitium with the same deposits.

The biopsy sample contained 45 glomeruli. On light microscopy, 15 glomeruli (33%) were globally sclerosed or severely hypoperfused. Discrete segmental expansion of the mesangial space by amorphous proteinaceous material was noted. Focal infiltration of the peritubular interstitium by amorphous, homogenous, acellular, and eosinophilic material was also noted. Approximately 50% of the sample showed extensive tubular atrophy and interstitial fibrosis. Arteries and arterioles showed the most striking finding: their walls were prominently infiltrated by the same amorphous eosinophilic material. The infiltrates caused severe narrowing of the vessels (Figure 1). 

**Figure 2:**
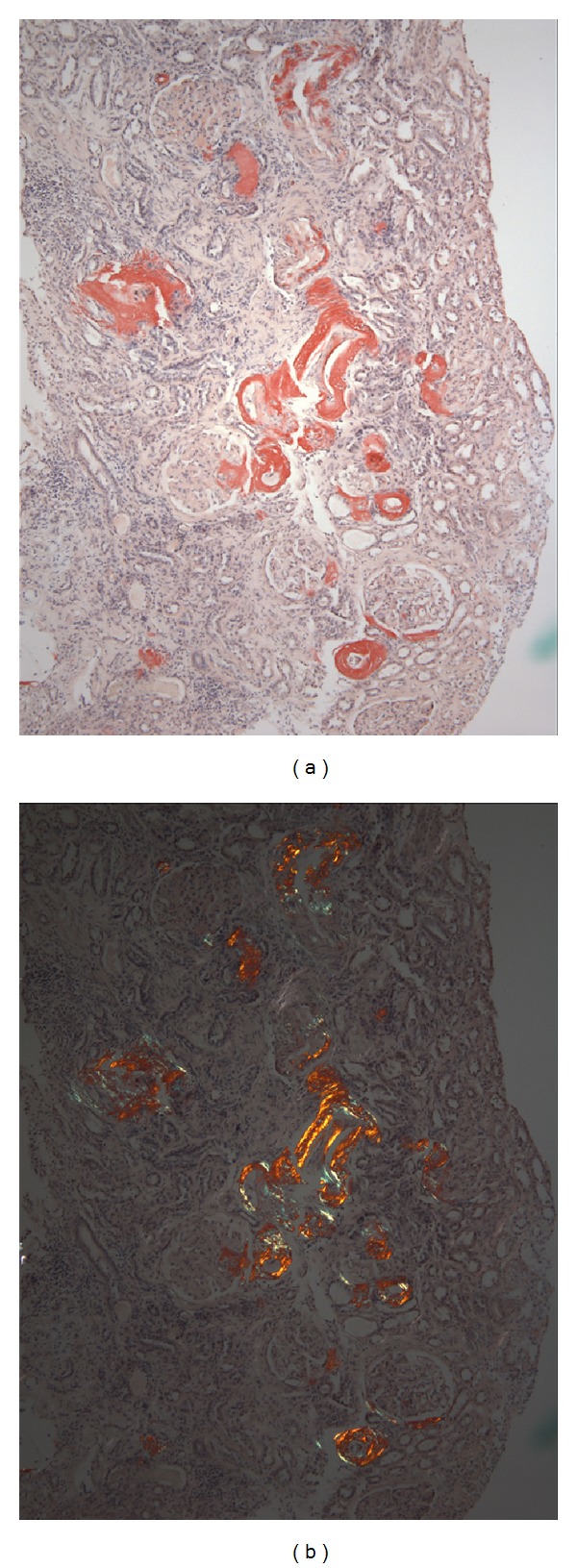
Congo red staining with red affinity primarily in the wall of the vessels and in the interstitium (a). The same structures show green birefringence on polarized light (b).

**Figure 3:**
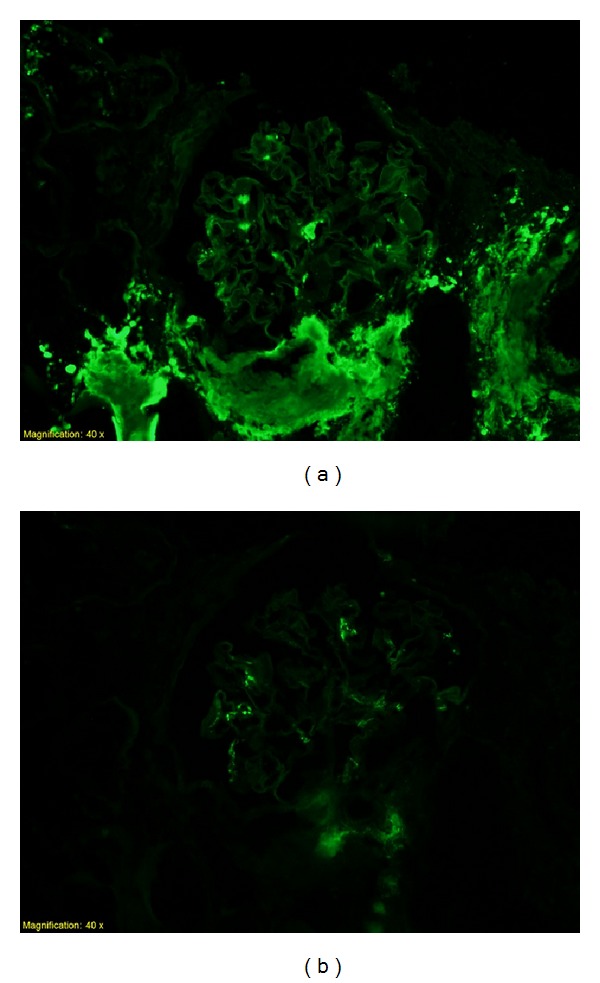
Immunofluoresence study showing reactivity of the amorphous eosinophilic material for kappa light chains predominantly in vessels, limited in glomerulusc (a), but not lambda light chains (b).

**Figure 4:**
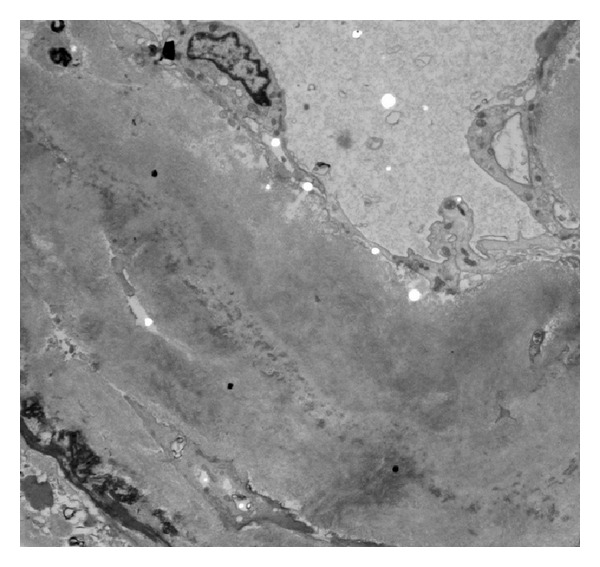
Electron microscopy picture showing a greatly thickened renal vessel wall with accumulation of fibrils.

**Figure 5:**
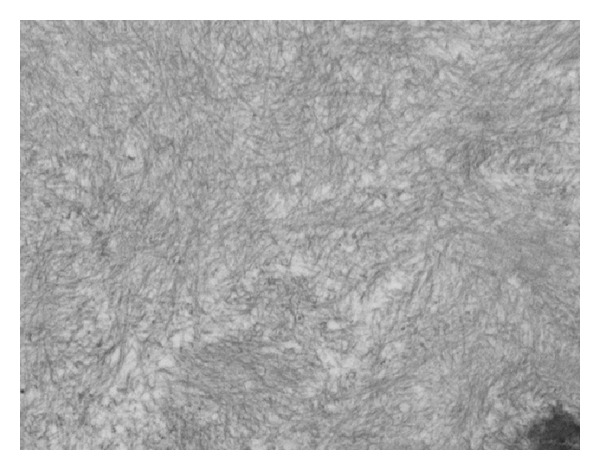
Higher magnification electron microscopy showing clearly the nonbranching fibrils in the wall of a renal vessel.

Congo red staining of the biopsy revealed red affinity of the amorphous eosinophilic material (Figure 2(a)) and green birefringence upon examination under polarized light (Figure 2(b)). Immunofluorescence revealed reactivity for kappa light chains (Figure 3(a)) but negative for lambda light chain (Figure 3(b)). The kappa light chain deposits were small, discrete and segmental in the glomeruli, coarse and irregular in the interstitium, and pronounced, coarse and confluent in the wall of the vessels (Figure 3(a)). Electron microscopy showed very small and segmental deposits of nonbranching fibrils of 11.5 nm width in the mesangial areas and the subepithelial spaces of glomeruli and market thickening of the wall of arterioles by extensive accumulation of these nonbranching fibrils (Figures 4 and 5). The final histologic diagnosis was AL amyloidosis, with reactivity for kappa light chains exclusively and affecting predominantly the vessels and the interstitium, and only very focally and segmentally the glomeruli.

Echocardiogram was not consistent with cardiac amyloidosis. He was started on melphalan and prednisone. However, his renal function progressively deteriorated, and hemodialysis was started in August 2012.

## 3. Discussion

A great variety of renal histological lesions has been associated with MGUS.  Table 1 shows histological patterns of renal disease associated with MGUS reported in the literature [6, 8–25]. This large variety of renal lesions associated with MGUS, the fact that the urinary findings may not conform to the expected findings in patients with a specific histological picture, which is illustrated by the patient of this report, plus the finding of renal pathology not associated with MGUS directly in some patients with MGUS and renal disease [6, 24], render kidney biopsy an indispensable tool for the diagnosis and management of renal disease complicating the course of MGUS. This point, however, has been established. 

The main point about AL amyloidosis secondary to MGUS illustrated by our patient is that the sites of deposition of amyloid in the kidneys are not always uniform and that clinical manifestations of renal disease are determined by the various sites of amyloid deposition. Moreover, this case presents with an uncommon deposition of kappa rather than lambda light chain. In addition to Bence Jones proteinuria, glomerular proteinuria, often in the nephrotic range, is a prominent manifestation of MGUS-related AL amyloidosis [6, 13]. The predominant site of AL amyloid deposition in the kidneys of patients with MGUS is the mesangium. In the study of Paueksakon et al. [6], all the 13 patients with MGUS and AL amyloidosis had mesangial expansion, and nephrotic syndrome was the most common clinical manifestation. Glomerular proteinuria is also a cardinal feature in patients with MGUS and other types of renal disease with primary glomerular involvement [7, 8, 11, 14, 16, 21–23]. 

Renal AL amyloidosis is not deposited exclusively in the glomeruli. Other kidney structures can be involved. In the study of Paueksakon et al. [6], AL amyloid deposits were also found in the renal interstitium in 8 of 13 patients and in the wall of the interlobular arteries in 7 of 13 patients. One patient with MGUS and AL amyloid deposits exclusively in the arteries had heavy proteinuria, but she also had glomerular immune-type deposits [15]. The glomerular deposits of AL amyloid were minimal in our patient, while the deposits in the wall of the arteries, and to a lesser extent, the interstitium, were prominent. Commensurate with the paucity of glomerular deposits was a persistent and complete absence of glomerular proteinuria. 

Predominant amyloid deposits in sites other than the glomeruli have been described in AA amyloidosis. AA amyloidosis involving primarily tubular basement membranes presents with a picture of interstitial nephritis [26], while AA amyloidosis involving primarily the renal vessels presents with minimal or no proteinuria and progressive renal failure [27, 28]. We propose that in addition to AA amyloidosis, AL amyloidosis developing during the course of MGUS may also in rare instances involve predominantly the renal arteries and arterioles and cause renal failure without proteinuria. The fibrils in AL amyloidosis are derived from the variable region of lambda light chains in approximately 75 percent of cases and kappa in the remainder [29]. 

In this case, the diagnosis of AL kappa amyloidosis and its sites of deposition in the kidney by a kidney biopsy may guide the choice of treatment.

## Figures and Tables

**Table 1:** Renal histological lesions during the course of MGUS.

Renal histology	References
Cryoglobulinemic glomerulonephritis	[6]
AL amyloidosis	[6, 13, 15, 24]
Light chain cast nephropathy	[6, 14, 24]
Light chain deposition disease	[6, 14, 19, 24]
Heavy chain deposition disease	[6, 14, 24]
Light chain and heavy chain deposition disease	[6, 14]
Light chain tubular crystal deposition	[6]
Waldenstrom's macroglobulinemic glomerulonephritis	[8]
Proliferative glomerulonephritis (several types)	[9, 24, 25]
Fibrillary or immunotactoid glomerulopathy	[10, 11, 16, 18]
Membranoproliferative glomerulonephritis	[12, 22]
Tubulointerstitial nephritis	[17]
Acute tubular necrosis	[20]
Membranous nephropathy	[21]
Dense deposit disease	[23]
Mixed lesions	[14, 15]
